# Neoadjuvant, adjuvant and palliative treatment of gastrointestinal stromal tumours (GIST) with imatinib: a centre-based study of 17 patients

**DOI:** 10.1038/sj.bjc.6600965

**Published:** 2003-07-29

**Authors:** P Bümming, J Andersson, J M Meis-Kindblom, H Klingenstierna, K Engström, U Stierner, B Wängberg, S Jansson, H Ahlman, L-G Kindblom, B Nilsson

**Affiliations:** 1 Department of Surgery, Lundberg Laboratory for Cancer Research, Sahlgrenska University Hospital, S-413 45 Göteborg, Sweden; 2 Department of Pathology, Lundberg Laboratory for Cancer Research, Sahlgrenska University Hospital, S-413 45 Göteborg, Sweden; 3 Department of Radiology, Lundberg Laboratory for Cancer Research, Sahlgrenska University Hospital, S-413 45 Göteborg, Sweden; 4 Department of Oncology, Lundberg Laboratory for Cancer Research, Sahlgrenska University Hospital, S-413 45 Göteborg, Sweden

**Keywords:** gastrointestinal stromal tumours, imatinib, *KIT* gene, prognosis, surgery

## Abstract

Malignant gastrointestinal stromal tumours (GIST) have a poor prognosis. Since these tumours are resistant to conventional radiation and chemotherapy, surgery has been the mainstay of treatment. However, surgery is usually inadequate for the treatment of malignant GIST. Imatinib, a KIT tyrosine kinase inhibitor, has recently been found to have a dramatic antitumour effect on GIST. In this centre-based study of 17 consecutive patients with high-risk or overtly malignant GIST, imatinib was used in three different settings – palliatively, adjuvantly, and neoadjuvantly. The treatment was found to be safe and particularly effective in tumours with activating mutations of exon 11 of the *KIT* gene. Clinical response to imatinib treatment correlated morphologically to tumour necrosis, hyalinisation, and reduced proliferative activity. The value of neoadjuvant imatinib treatment was illustrated in one case.

Gastrointestinal stromal tumour (GIST), the most common mesenchymal tumour of the alimentary tract, has a wide clinical spectrum and highly variable prognosis. The outcome of malignant GIST is extremely poor (DeMatteo *et al*, 2000). Since GIST is resistant to conventional chemotherapy and radiation, complete surgical resection has been the only available treatment. However, surgery is often inadequate for advanced GIST.

GIST has been found to differentiate towards a pacemaker cell phenotype and to express the KIT tyrosine kinase receptor uniformly (Hirota *et al*, 1998; Kindblom *et al*, 1998). In addition, they frequently exhibit activating mutations of the *KIT* proto-oncogene (Hirota *et al*, 1998; Lasota *et al*, 1999; Sakurai *et al*, 1999; Taniguchi *et al*, 1999; Rubin *et al*, 2001; Andersson *et al*, 2002). Such activating *KIT* gene mutations are potential targets for therapeutic intervention with imatinib, a novel competitive inhibitor of a family of tyrosine kinase receptors including BCR-ABL, ABL, KIT, and PDGF-R.

Joensuu *et al* (2001) have recently reported the first successful treatment of a patient with metastatic GIST using imatinib. Preliminary results of several trials indicate that imatinib is an effective and safe treatment in patients with unresectable and/or metastatic GIST (Van Oosterom *et al*, 2001; Demetri *et al*, 2002). We report the results of a consecutive series of 17 patients with high risk and overtly malignant GIST, who were treated at our centre using imatinib in palliative, adjuvant, and neoadjuvant settings.

## PATIENTS, TUMOURS, AND METHODS

### Patients

Patients with ‘high-risk’ (Fletcher *et al*, 2002) or overtly malignant GIST (metastatic disease at presentation), who were admitted to Sahlgrenska University Hospital from May 2001 to September 2002, were included in this study (*n*=17, Table 1

**Table 1:** Clinical data and tumour characteristics at the time of initial diagnosis and imatinib induction, and response to imatinib in 17 patients with malignant GIST

	**Status at initial diagnosis**					**Status at imatinib induction**			**Imatinib treatment**				
**Case**	**Age/sex**	**1° tumour site and size (cm)**	**Ki67%/MR**	**Exon 11 KIT mutation**	**Mets at initial diag.**	**Number previous surgeries**	**Interval: 1° tumour diag to imatinib (months)**	**Disease status**	**Clinical setting**	**FU (months)**	**Response CT eval.**	**Response PET eval.**	**Side effects**
1	56/M	SB/20	10%/2–5	W557R	L	None	3	PT, P, L	Neoadjuvant	10	PR	No residual uptake	None
2	48/M	R/9	10%/2–5	K558_V560del	None	1	1.5	NED^a^	Adjuvant	13	NED	No uptake	None
3	55/M	R/4.5	5%/<2	W557M	None	2	180	NED^b^	Adjuvant	12	NED	Not done	None
4	10/F	S/3.5	5%^c^/2–5	None	None	5	144	NED^d^	Adjuvant	9.5	NED	No uptake	Oedema grade 2
5	74/M	D/11	15%/6–10	None	None	2	22	P, L	Palliative	13	PD	Additional uptake	Oedema grade 1
6	70/M	D/25	25%/6–10	None	L	1	1	PT, L	Palliative	9	PD	Not done	None
7	47/M	SB/29	30%/>10	W557_E561del	P	1	7	PT, P, L	Palliative	14.5	PR	No residual uptake	None
8	63/M	SB/15	50%/2–5	None	P, L	2	3	P, L	Palliative	9	PR	Decreased uptake	None
9	70/M	SB/16	20%/>10	V559_E561del	P, L	3	14	P, L	Palliative	18	PR	Not done	None
10	72/M	SB/12	10%/>10	N564_L576del	P, L	1	3	P, L	Palliative	9.5	PR	Not done	None
11	53/M	SB/6	10%/>10	P551del,M552L	L	1	4	L	Palliative	14.5	PR	Not done	Oedema grade 2
12	62/M	SB/5	10%/>10	None	None	1	16	Pu	Palliative	9	SD	Not done	None
13	56/M	D/6	5%/6–10	K558_V559del, V560I	None	5	84	P, L	Palliative	12.5	PR	Not done	Oedema grade 1
14	68/F	SB/10	10%/6–10	V559del	None	2	12	L	Palliative	6	PR	No residual uptake	None
15	36/M	SB/10	5%/2–5	W557_K558del	P	1	4	NED	Adjuvant	7	NED	No uptake	None
16	62/M	R/15	15%/6–10	P551_E554del	None	1	2	NED^a^	Adjuvant	7	NED	No uptake	None
17	74/M	R/30	25%/>10	K558S,V559del	P	1	9	P	Palliative	8	PR	No residual uptake	None

D=duodenum; del=deletion; diag.=diagnosis; eval.=evaluation; F=female; FU=follow-up; L=liver mets; M=male; mets=metastases; MR=mitotic rate (mitoses/50 h.p.f.); NED=no evidence of disease; P=peritoneal mets; PD=progressive disease (>20% increase in the greatest diameter of the target lesion or appearance of one or more new lesions); PR=partial response (>30% decrease in the greatest diameter of target lesion); PT=primary tumour; Pu=pulmonary mets; R=rectum; S=stomach; SB=small bowel; SD=stable disease; 1°=primary;

a Resection of primary rectal GIST with intralesional margins.

b Resection of local recurrence with intralesional margins.

c Focally 25% of cells positive for Ki67.

d After five surgical procedures for peritoneal mets and liver mets.

). Clinical data at the time of initial diagnosis and at the initiation of imatinib treatment are detailed in Table 1. Patient 1, who was given imatinib neoadjuvantly, is described in detail in the next section. Three of the five patients (cases 2, 3, and 16) who were treated with adjuvant imatinib had been surgically treated for rectal GIST: two were primary tumours and the other a local recurrence. None of these three patients had clinical or radiographic evidence of residual tumour following surgery; however, microscopic examination revealed that all had intralesional surgical margins. A fourth patient (case 15) with a small bowel GIST believed to be completely surgically removed also had intralesional margins microscopically. The fifth patient (case 4) who received adjuvant treatment had a gastric GIST. This patient had undergone five surgical resections for peritoneal and liver metastases during a 12-year period prior to receiving adjuvant imatinib. No residual tumour was detected in this patient following the last surgery. Of 11 patients who received palliative treatment, 10 had an unresectable primary tumour peritoneal metastases and/or liver metastases at the time of imatinib induction; the remaining patient had pulmonary metastases. Five of the 11 patients who received palliative imatinib had two to five surgical resections of tumour prior to receiving imatinib. The time interval from initial diagnosis of GIST to induction with imatinib is given in Table 1.

All resected or biopsied primary tumours, recurrences, and metastases were histologically reviewed. Histologically, three tumours were predominantly epithelioid, eight predominantly spindled, and six mixed spindled and epithelioid. The number of mitotic figures per 50 consecutive high-power fields (h.p.f.= 0.113 mm^2^) was recorded as <2, 2–5, 6–10, and >10/50h.p.f.; proliferative activity was assessed visually estimating the percentage of Ki67-immunopositive tumour cells using MIB1 (see Table 1). Immunostains for CD117, CD34, *α*-smooth muscle actin, desmin, and S100 protein were performed in all 17 cases and found to be positive in 17, 14, two, zero, and zero cases, respectively.

Treatment with imatinib (400 mg p.o. once daily) was given neoadjuvantly (*n*=1), adjuvantly (*n*=5), or palliatively (*n*=11) following recommendations given by Van Oosterom *et al* (2001) and side effects were monitored in agreement with the US National Cancer Institute Common Toxicity Criteria (Version 2.0) (Cancer Therapy Evaluation Program, 1999). Objective tumour response was evaluated by spiral computed tomography (CT) (5 mm contiguous reconstruction algorithm) 3, 6, and 12 months after initiating treatment with imatinib and categorised according to the RECIST (Therasse *et al*, 2000). Magnetic resonance imaging (MRI) (contiguous cuts of 10 mm or less) was performed in the patient who received neoadjuvant therapy (case 1). Positron-emission tomography (PET) with [^18^F]fluorodeoxyglucose (^18^F-FDG) as tracer was performed before and during treatment with imatinib in 10 patients (Table 1).

### RNA and DNA isolation and nucleotide sequence analyses

RNA and cDNA were prepared from fresh-frozen tissues in nine cases (cases 1–9) as previously described (Andersson *et al*, 2002). Genomic DNA was prepared from three 5-*μ*m-thick sections of paraffin-embedded tumour material in eight cases (cases 10–17) using the DEXPAT kit (TAKARA, Kyoto, Japan). Exon 11 of the *KIT* gene was amplified by PCR and directly sequenced in all cases as well as cloned and sequenced in 10 cases (cases 2, 3, 7, 9–11, 13–15, 17) as previously described (Andersson *et al*, 2002) using primers PCRKIT21 s and PCRKIT22as for cDNA and primers PCRKIT31 s and 34as for genomic DNA. Primer sequences are shown in Table 2

**Table 2:** Primer sequences

**Primer name**	**Primer sequence**
PCRKIT21s	5′-ACTCCTTTGCTGATTGGTTTCGT-3′
PCRKIT22as	5′-AATGGTGCAGGCTCCAAGTAGAT-3′
PCRKIT31s	5′-CCAGAGTGCTCTAATGACTG-3′
PCRKIT34as	5′-CTGTTATGTGTACCCAAAAAGG-3′
Sp6s	5′-ATTTAGGTGACACTATAG-3′
T7as	5′-TAATACGACTCACTATAGGG-3′

s=sense; as=antisense.

.

## RESULTS

### Imatinib treatment

The results of imatinib treatment results are summarised in Table 1. Results regarding case 1 are described in detail below. None of the five patients who received adjuvant treatment have had recurrent disease during 7–13 months of follow-up. Eight of 11 patients who received palliative imatinib treatment have had a partial response (defined as >30% decrease in the largest dimension of the target lesion on CT) over a follow-up period of 6–18 months. One of the 11 patients has had stable disease. Two patients in whom no *KIT* mutations were detected (cases 5 and 8) died of myocardial infarcts 9 and 13 months, respectively, after imatinib induction. Both patients had progressive tumour disease at the time of death.

None of the 17 patients in this study have developed serious side effects. Notably, there was neither intra-abdominal nor gastrointestinal haemorrhage in any patient. Four patients developed transient oedema, grade 1–2.

### Case report (patient 1), neoadjuvant imatinib treatment

**Figure 1:**
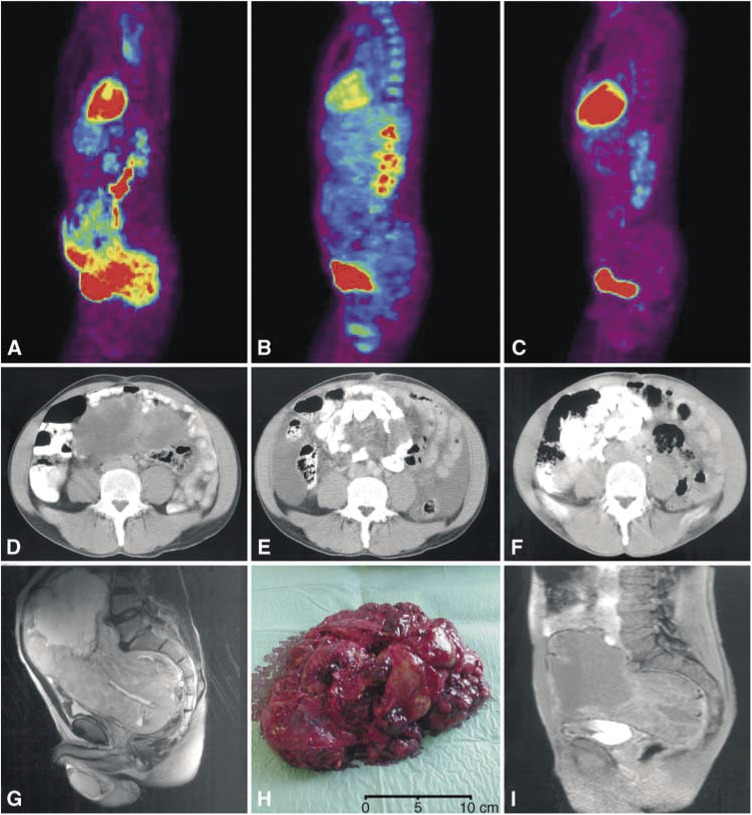
Case 1, neoadjuvant imatinib treatment. Positron-emission tomography examinations before (**A**), after 3 weeks (**B**), and after 12 weeks (**C**) of imatinib treatment. The increased uptake of the tracer [^18^F] fluorodeoxyglucose that was seen in a huge abdominal tumour before treatment cannot be detected at PET examinations after 3 and 12 weeks of imatinib treatment. Corresponding CT examinations before (**D**), after 3 weeks (**E**), and after 12 weeks of imatinib treatment (**F**). During 12 weeks of treatment, the tumour decreased from 35 to 18 cm. Corresponding sagittal gadolinium-enhanced T_2_-weighted and T_1_-weighted MRI before (**G**) and after 12 weeks (**I**) of treatment, respectively, showing tumour reduction. After 3 months of treatment, the tumour could be completely excised, leaving the rectum intact (**H**).

A 56-year-old man was admitted with a palpable, huge tumour filling the lower abdomen. Computed tomography and MRI examinations revealed a 35 cm tumour, occupying most of the pelvis and lower abdominal cavity in addition to mesenteric metastases and six liver metastases involving both lobes of the liver. Core needle biopsies of the intra-abdominal tumour and the liver metastases were diagnosed as a malignant spindled and epithelioid GIST. Mutation analysis of core needle samples revealed a substitution of tryptophan to arginine in amino-acid position 557 (W557R) of exon 11 of the *KIT* gene. Positron-emission tomography scan showed increased uptake in the intra-abdominal tumour and all liver metastases. Both the primary tumour and the liver metastases were considered unresectable. Imatinib treatment was therefore initiated. Positron-emission tomography scan was repeated after 3 and 12 weeks; no tracer uptake was seen at either examination. After 3 weeks of imatinib treatment, a marked decrease in tumour size was seen on CT examination. After 12 weeks, the tumour had decreased from 35 to 18 cm in greatest dimension, and all six liver metastases had become entirely cystic. Since the primary tumour was now considered to be resectable, complete surgical excision of the tumour (found to originate from the ileum) and multiple mesenteric metastases were performed leaving the rectum, bladder, and prostate intact (Figure 1A–I

). The patient is still on the same dose of imatinib without side effects 7 months after surgery. Positron-emission tomography scan shows no residual uptake and CT demonstrates a stable partial response (lesions with low attenuation).

### Morphologic response to imatinib treatment

**Figure 2:**
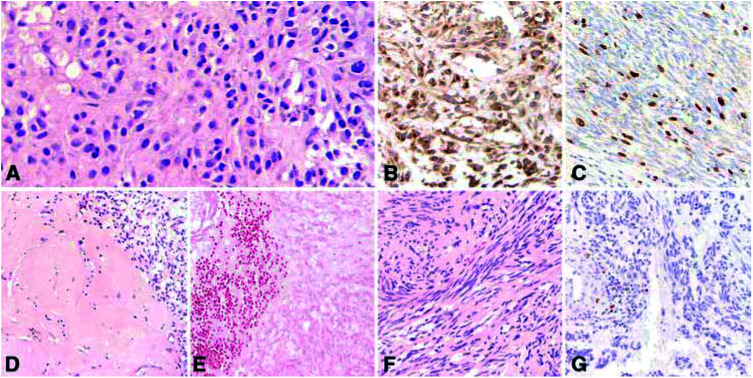
Case 1, neoadjuvant imatinib treatment. Pretreatment core needle biopsies of a huge abdominal tumour and liver metastases showing characteristic features of a malignant spindled and epithelioid GIST (**A**) that is immunoreactive for CD117 (**B**) and MIB1 (Ki67 proliferative index=10%) (**C**). After 3 months of imatinib treatment, the tumour shows extensive hyalinisation with scattered residual tumour cells (**D**), necrosis (**E**), cyst formation and areas of viable tumour (**F**) in which the proliferative index is virtually zero (only a few inflammatory cells next to a vessel are MIB1 immunoreactive, **G**).

Tumour tissue from three patients (cases 1, 5, and 8) was studied during imatinib treatment. The tumour of the patient, who received 3 months of neoadjuvant imatinib treatment (case 1) showed extensive areas of tumour necrosis, hyalinised areas with sparse, scattered tumour cells containing small, condensed nuclei and areas of viable tumour histologically similar to those seen in pretreatment biopsies. Prior to imatinib treatment, the tumour revealed a mitotic rate of 5/50 h.p.f. and a 10% proliferative index compared to a post-treatment mitotic rate of 0/50 h.p.f. and a proliferative index far less than 1%. (Figure 2A–G

). In case 5, the mitotic rate decreased from 8 to 5/50 h.p.f. and the proliferative index from 15 to 5% after 10 months of treatment, whereas no change in either parameter was seen in case 8 after 7 months of treatment.

### KIT mutation analysis

Typical gain-of-function exon 11 *KIT* mutations were found in 12 of 17 patients, including two point mutations (cases 1 and 3), three point mutations and deletions (cases 11, 13, and 17), and seven deletions (cases 2, 7, 9, 10, 14, 15, and 16) (see Table 1). Eight of nine patients with a partial response had an exon 11 mutation. One patient with a partial response (case 6), two patients with progressive disease (cases 5 and 6), and one patient with stable disease (case 12) had no exon 11 *KIT* mutations. Four of the five patients who received adjuvant treatment had an exon 11 mutation.

## DISCUSSION

This report indicates the utility of neoadjuvant imatinib treatment in downstaging selected patients with malignant GIST, thereby rendering the primary tumours and metastases resectable in some cases. As illustrated in case 1, preoperative imatinib treatment during a 12-week period led to dramatic reduction of tumour size, allowing complete removal of the tumour.

Eight of nine patients with a partial response to imatinib, including the patient treated neoadjuvantly, had activating exon 11 *KIT* mutations. In contrast, none of the three patients with progressive or stable disease had exon 11 mutations. These results agree with a recent report demonstrating a significantly higher response rate to imatinib among patients with GIST containing exon 11 *KIT* mutations compared to those without such mutations (Heinrich *et al*, 2002a, 2002b). Interestingly, one patient in our series (case 8) whose tumour lacked an exon 11 *KIT* mutation had a partial response to imatinib by CT and a significantly reduced tracer uptake on PET. The tumour of another patient with progressive disease (case 5) also lacked an exon 11 mutation and showed a significant reduction in proliferative activity after 10 weeks of imatinib treatment. These observations as well as those of Heinrich *et al* (2002a, 2002b) suggest that activating *KIT* mutations may not necessarily be a prerequisite for imatinib treatment.

The morphologic findings of extensive sclerosis and hyalinisation with scattered, shrunken tumour cells replacing previously viable tumour after 3 months of neoadjuvant imatinib (case 1) are in agreement with those reported by Joensuu *et al* (2001). In this case and another (case 5), we also observed foci of highly cellular, viable tumour after imatinib treatment, similar to those seen in pretreatment biopsies but with dramatically reduced proliferative activity. These findings may indicate tumour heterogeneity. They also correspond with reports that imatinib occupies the ATP-binding site of the target kinase receptor and prevents subsequent autophosphorylation, thereby leading to the onset of apoptosis and decreased proliferation (Goldman and Melo, 2001; Tuveson *et al*, 2001).

The recent development of imatinib has drastically improved the limited therapeutic options and the dismal outlook for patients with advanced, malignant GIST. Additional studies are needed to establish the long-term effectiveness of imatinib treatment and to further define treatment strategies combining imatinib and surgery. We believe that surgery will continue to play a crucial role in the treatment of malignant GIST.

In conclusion, this study illustrates several new and interesting aspects regarding imatinib treatment in malignant GIST, including a role for neoadjuvant treatment that may allow surgical resection of large primary tumours and metastases; a high response rate to imatinib in patients whose tumours have exon 11 *KIT* mutations; a potential role for imatinib even in patients without exon 11 mutations; and histologic documentation of different tumour responses to imatinib corresponding clinically to reduction in tumour volume. Our results also indicate a role for adjuvant treatment with imatinib, which is currently being investigated in prospective randomised clinical trials.
